# Aetiology, Epidemiology, Histopathology, Classification, Detailed Evaluation, and Treatment of Ovarian Cancer

**DOI:** 10.7759/cureus.30561

**Published:** 2022-10-21

**Authors:** Udit M Zamwar, Ashish P Anjankar

**Affiliations:** 1 Pathology, Jawaharlal Nehru Medical College, Datta Meghe Institute of Medical Sciences, Wardha, IND; 2 Biochemistry, Jawaharlal Nehru Medical College, Datta Meghe Institute of Medical Sciences, Wardha, IND

**Keywords:** differential diagnosis, aetiology, histopathology, risk factors, epidemiology, ovarian cancer

## Abstract

There are a minimum of five distinct sub-types of ovarian cancer based on histology, each of which has distinct factors of risk, types of cells, molecular makeups, clinical characteristics, and therapeutic approaches. Ovarian cancer is detected usually at later stages, and there is no reliable screening method. Cytoreductive surgery and chemotherapy which use platinum-containing drugs are the standard treatments used for freshly detected cancer. Chemotherapy, drugs that are anti-angiogenic, poly ADP-ribose polymerase inhibitors, and immunological treatments are all used to treat recurrent cancer. The most frequent type of ovarian cancer to be diagnosed is high-grade serous carcinoma (HGSC), which often responds well to platinum-based chemotherapy when discovered. However, HGSCs commonly relapse and develop increased treatment resistance in addition to the other histologies. As a result, ovarian cancer research is actively focused on understanding the processes causing platinum resistance and developing strategies to combat it. Serous tubal intraepithelial carcinoma is an HGSC precursor lesion. It is one of the early complications seen in ovarian carcinoma. It has been very useful in identifying the people who have a greater chance of developing ovarian cancer and development of strategies to prevent it. This has led to a significant progress for identification of the genes which are found in people with greater chances of development of ovarian carcinoma (for example, the BRCA1 and BRCA2).

## Introduction and background

Ovarian cancer is one of the gynecological cancers that is detected all over the world. It is known to have a high rate of mortality in most people suffering from it. It is the fifth most common cause of death in the female population all over the globe [1]. This cancer is extremely hard to diagnose in the early stage and mostly gets detected in advanced stages, which leads to it not being properly managed. The screening tests which are used at present are not very useful in the detection of this cancer. The keys to the detection of this cancer are vaginal ultrasound and a biomarker known as CA-125, but they too have failed to prevent the high rates of deaths caused by this cancer [2].

The preferred treatment for this is undergoing chemotherapy which use cisplatin as the drug of choice. Anti-angiogenic bevacizumab, along with poly ADP-ribose polymerase inhibitors, has been a bit more successful in curing this cancer at present [3].

This cancer is known to recur at a tremendous rate after the initial treatment. The case that undergoes relapse mostly has very less chances of being cured and the treatment given also fails to have the desired effect. There is a need for effective prevention and diagnostic techniques to prevent this cancer. In this article, the epidemiology, along with the factors causing risk of ovarian cancer, are reviewed. This article also showcases the evaluation and multi-disciplinary approach in managing this condition along with some of the newest trials which are currently in progress.

## Review

Aetiology

The precise cause of ovarian cancer is not known.There are numerous factors that facilitate increasing the chances of the development of ovarian cancer. Lifestyle factors such as cigarette smoking, obesity, and unhealthy diet may affect the risk of developing ovarian cancer. Exposure to certain environmental agents such as talc, herbicides, and pesticides may cause an increase in risk of ovarian cancer. However, lifestyle and environmental factors have a very minimal role in the development of ovarian cancer. It is most commonly seen in women after menopause, where the age increment is related to an increase in the number of cases, advancement in the stage of this disease, and very few cases of survival [4]. The factor which is the biggest cause of carcinoma of the ovary is having a history of ovarian or breast cancer in the family [5]. Mutations in BRCA (BRCA1 and BRCA2) genes are also one of the major causes of ovarian cancers. Some studies on ovarian carcinoma also suggest that repeated ovulation may also be a cause of increasing the risk of developing ovarian cancer. Loss of p53 gene which is a tumor suppressor gene may also cause an increase in risk of ovarian cancers. This is a molecular mechanism and about 55% of women with ovarian cancer where found to be lacking the p53 gene [4].

Epidemiology

In the year 2020, there are an approximately 21,400 new cases of carcinoma of the ovary, which estimate to be 1.2% of all the cases of cancer. The mortality related to it is 13,700. There is a 47.3% chance of survival for five years for women. Approximately 13.7% of cases of ovarian cancer are detected at a local stage, and about 52% of cases are detected at the metastasized stage, in which the survival rate of five years gets as low as 29.7% instead of 91.8% if diagnosed earlier before spreading locally. 90% of ovarian carcinomas are of the epithelial type, with the most common as the serous sub-type. Rates adjusted as per age of new cases of carcinoma of the ovary are decreasing in popularity based upon the models of analysis which are statistical [6].

Histopathology and classification

Histopathology

The four most common types of carcinoma of the ovary which are of the epithelial type according to histology are serous, clear-cell, endometrioid, and mucinous tumor. They are divided further into sub-types on the basis of their peculiar biology and response to treatment. The sub-types which are not common are Brenner and seromucous.

Ovarian carcinoma is divided into two subtypes: Type 1 and Type 2 tumors, of which Type 2 is more fatal, and the causative factor for it is continuous cycles of ovaries, which lead to inflammation and endometriosis. Type 1 tumor includes the endometrioid, serous type of low-grade, clear-cell, and mucinous cancers, of which the rarest sub-types are seromucous and Brenner tumors. Type 1 tumors are most probably caused by atypical proliferative (borderline) tumors. Type 2 tumors consist of high-grade carcinoma of serous type, carcinosarcoma, and carcinoma, which are not differentiated and generally originate from tubal intra-epithelial carcinoma of serous type. Type 1 tumors are present in the early stages usually and are low grade, the exception being clear-cell tumor, which is considered high grade. They proliferate usually at a slow rate. Their diagnosis is early and also has a good rate of prognosis. When both tumors are compared, the Type 2 tumors are classified as high-grade tumors and are mostly diagnosed at a stage that is advanced in nature. They have a very extreme rate of proliferation with a fast and aggressive progress rate and instability of a very high degree in chromosomes as compared to Type 1 which has p53 mutations being present mostly in the cases [7].

Ovarian cancer of the serous type is the most common sub-type of ovarian carcinoma. It usually is seen as low grade (10% of every tumor of serous sub-type) or as high-grade cancer (90% of every tumor of serous sub-type). The low-grade serous carcinoma (LGSC) shows minimum atypia in the nucleus, mitosis very rarely, and the molecular defects are also very less. In comparison, the high-grade serous carcinoma (HGSC) presents nuclear atypia more significantly and mitosis with more molecular defects as viewed by the use of cytogenetic analysis [8]. LGSCs mostly are detected in younger age groups of people and have a more good prognosis as compared to the HGSCs, which are likely to be diagnosed in older age groups of people and also have a 10-year rate of mortality of 70% [9].

Only 5% of ovarian carcinomas are clear-cell ovarian cancers, which are less common. They exhibit cellular clearance, a growth pattern of cystic type, and a typical hobnail development pattern histologically as well as pathologically. According to immunohistochemistry, Stage 1 and Stage 2 cancers are predominately overexpressed with BAX, whereas primary lesions are overexpressed with BCL-2, an anti-apoptotic protein. In the early stages of ovarian cancer, the clear cell type tumors had a BCL-2/BAX ratio smaller in comparison with the metastatic lesions, which have a greater relative ratio [10]. They are frequently detected in earlier stages and, like endometrioid tumors, have a favourable outlook.

All serous ovarian cancers exhibit high and diffuse staining for cytokeratin-7 (CK7). In 80% to 100% of mucinous ovarian tumors, it is positive, and other ovarian epithelial cancers also exhibit CK7 positivity. In contrast to metastatic colorectal cancer, which has a positivity rate of about 25%, about 96% of adenocarcinoma of the ovary tested positive for CK7 [11].

*WHO Classification of Ovarian Cancer* 

The WHO has classified ovarian cancer into the following types: 1) Epithelial tumors, 2) Mesenchymal tumors, 3) Mixed epithelial and mesenchymal tumors, 4) Sex-cord stromal tumors, 5) Germ cell tumors, 6) Monodermal teratoma and somatic type tumors arising in dermoid cyst, 7) Miscellaneous tumors, 8) Mesothelial tumors, 9) Soft tissue tumors, 10) Tumor-like lesions, 11) Lymphoid/myeloid tumors, and 12) Secondary tumors.

Epithelial tumors are further classified into: 1) Serous tumors (cystadenoma, adenofibroma, surface papilloma, and borderline tumor), 2) Mucinous tumors (cystadenoma, adenofibroma, borderline tumor, and carcinoma), 3) Endometriod tumors (cystadenoma, adenofibroma, borderline tumor, and carcinoma), 4) Clear-cell tumors (cystadenoma, adenofibroma, borderline tumor, and carcinoma), 5) Seromucinous tumors (cystadenoma, adenofibroma, and borderline tumor), and 6) Brenner tumors (borderline and malignant) [7]. Mesenchymal tumors are further classified into: 1) Endometriod stromal sarcoma (low grade and high grade), 2) Smooth muscle tumors (leiomyoma and leiomyosarcoma), and 3) Ovarian myxoma. Mixed epithelial and mesenchymal tumors are further classified into adenocarcinoma.

Sex cord stromal tumors are further classified into: 1) Pure stromal tumors (fibroma, thecoma, sclerosing tumor, microcystic tumor, signet ring tumor, leydig cell tumor, steroid cell tumor, and fibrosarcoma), 2) Pure sex cord tumors (adult granulosa cell tumor, juvenile granulosa cell tumor, sertoli cell tumor, and sex cord tumor with annular tubules), and 3) Mixed sex cord stromal tumors (sertoli-leydig cell tumor, sex cord stromal tumor, and gynandroblastoma) [7]. Germ cell tumors can be further classified into: 1) Benign teratoma, 2) Immature tertoma, 3) Dysgerminoma, 4) Yolk sac tumor, 5) Embryonal carcinoma, 6) Choriocarcinoma, 7) Mixed cell tumor, 8) Monodermal teratoma and somatic type tumors arising from a dermoid cyst, and 9) Germ cell sex cord stromal tumors.

Miscellaneous tumors can be further classified into: 1) Rete cystadenoma/adenoma, 2) Wolffian tumor, 3) Solid pseudopapillary tumor, 4) Small cell carcinoma of the ovary, and 5) Wilms tumor. Tumor-like lesions can be further classified into: 1) Follicle cyst, 2) Corpus luteum cyst, 3) Large solitary luteinized follicle cyst, 4) Hyperreactio luteinalis, 5) Pregnancy luteoma, 6) Stromal hyperplasia and hyperthecosis, 6) Fibromatosis and massive edema, and 7) Leydig cell hyperplasia.

History and clinical examination

Ovarian cancer symptoms are non-specific, making it simple to overlook them at earlier stages as they can be attributed to any other disease processes of the same potential. Frequently, the symptoms do not show up until a late stage (Stage 3 or Stage 4). Feeling of fullness of abdomen, bloating, nausea, distention of the abdomen, early satiety, exhaustion, changes in bowel habits, urine symptoms, pain in the back, dyspareunia, and loss of weight are among the symptoms that present. Uncertain months pass before the detection of cancer of the ovary until the symptoms appear [12].

In clinical situations which are highly suspicious, performing an examination is a must, including a rectovaginal examination on a bladder that is empty to search for tumors in the abdominal and pelvic region. In more severe situations, we can find a mass in the pelvic region which is palpable, ascites, or reduced breath sounds because of pleural effusions. It is uncommon to see a sister Mary Joseph nodule because of metastases to the umbilicus. A clinical hint pointing to the existence of cancer that is concealed is provided by the Lesar-Trélat sign, which is defined as a sudden increment in the discovery of seborrheic keratosis [13].

Rarely can paraneoplastic disorders be linked to ovarian cancer. Ovarian cancer and Trousseau's syndrome have been linked with each other. Increment in the levels of the hormone-releasing protein from the parathyroid gland may cause increased levels of calcium in the blood, which can cause symptoms like disturbed mental condition, exhaustion, constipation, pain in the abdomen, the patient feels more thirst and frequent urination [14].

Evaluation

Transvaginal ultrasonography (extremely sensitive and preferable) and/or abdominal and pelvic ultrasonography is performed on patients who have a high level of clinical suspicion. It provides a good idea of the ovarian mass size, location, and complexity. Additional imaging techniques, such as pelvic MRI, PET scan, and/or chest and abdominal pelvis CT scan, can be used to define tumor expansion.

The imaging is typically done in conjunction with the measurement of CA-125 levels. Most epithelial ovarian tumors have increased CA-125 levels overall, while only 50% of those with early-stage epithelial ovarian cancers have such increased levels of CA-125 [15]. It has been discovered that postmenopausal people had higher specificity and positive predictive value than premenopausal people. The other pathological disorders whether benign or physiological which include endometriosis, pregnancy, cysts in the ovary, and inflammatory peritoneal illnesses are also associated with the increased CA-125 levels. As a result, research on additional biomarkers is now being done to increase the specificity of ovarian cancer biomarkers. A novel biomarker called human epididymis protein 4 (HE4) is being studied right now. Around 100% of serous and endometrioid subtypes have it, making it more vulnerable to ovarian cancer. High amounts of CA-125 and HE4 are the markers of malignant tumors in the ovaries and may one day be used as a diagnostic tool, according to recent studies [16]. The risk of malignancy index (RMI), which also takes into account the findings of transvaginal ultrasonography and the status of menopause in people, can be computed using CA-125 levels. RMI greater than 200 is more than 96% specifically related to a high risk of malignancy [13].

The risk of developing cancer is calculated using the risk of ovarian malignancy algorithm (ROMA), which makes use of a mathematical calculation that integrates HE4 and CA-125 values that have been adjusted according to the status of menopause [17]. The ROMA is a test done for screening and is also of high benefit that makes use of the high specificity and high sensitivity of HE4 and CA-125 respectively to identify more ovarian cancer cases overall, particularly in the early stages.

It involves performing an exploratory laparotomy, closely examining the abdomen and pelvic regions for disease, and checking the peritoneal surfaces with a biopsy or pelvic washings as necessary. The International Federation of Gynaecology and Obstetrics (FIGO) is responsible for creating the classification of stages of ovarian carcinoma [18]. Bilateral salpingo-oophorectomy (BSO), along with dissection of lymph nodes of the pelvic and the para-aortic region, is performed after total abdominal hysterectomy. The ultimate diagnosis of the histological type, grade, and stage is based on the tissue biopsies examined by a pathologist [9].

Treatment

Debulking Surgery

Chemotherapy and surgery are typically made use of in the standard therapy of ovarian carcinoma. When the lesions have very low chances of developing malignancy in the early stages of invasive epithelial ovarian cancer, a unilateral salpingo-oophorectomy is conducted. In this operation the uterus and the normal ovary are not removed [19]. A debulking procedure involving a hysterectomy and BSO has demonstrated better results for advanced-stage ovarian cancer. Exploratory laparoscopic surgery must be performed first to ascertain if the debulking surgery would be advantageous for diseased people. Large or persistent tumor burdens can prevent blood flow to the affected area, causing tissue damage and raising the risk of further cellular damage and multi-drug treatment resistance [9]. Compared to debulking surgeries, laparoscopic procedures are known to be less intrusive and require a shorter recovery period. The women suffering from ovarian cancer should undergo somatic and germline testing as well as genetic risk assessment if they have not already, as the genetic risk assessment is the informant of maintenance therapy [20].

Maximal Cytoreductive Surgery

Maximal cytoreduction is included among the most potent independent predictors of enhanced median survival in people suffering from Stage 3 or 4 ovarian cancer. Therefore, optimal cytoreduction has been highly advised to gain no residual disease, regardless of the sequence of the performed operation, whether it occurs before the neoadjuvant chemotherapy or after it. A meta-analysis of 6,880 diseased women suffering from Stage 3 and Stage 4 ovarian cancer revealed a median overall survival increase of 5.5% and a maximal cytoreduction rise of 10% in one of the studies [21]. There was a 50% improvement in mean-weighted median survival time when actuarial survival was computed between cohorts that had lesser than or equal to 25% maximal cytoreduction and more than 75% maximal cytoreduction. However, there was no statistically significant correlation between the platinum dose intensity and the log-median survival time [22]. In order to ensure early surgical intervention in the disease course, interval cytoreduction surgery is typically done after four cycles or less of neoadjuvant chemotherapy. However, there should be at least 20 days between the patient's initial neoadjuvant chemotherapy regimen and any surgical intervention because there is a danger of severely reduced postoperative healing if the patient had bevacizumab as part of it [23].

Primary Chemotherapy and Neoadjuvant Therapy

Neoadjuvant chemotherapy has been thoroughly researched and is supported by the evidence when given to people with early-stage ovarian carcinoma. Every patient must have their own unique clinical decision made in the end. Patients suffering from early-stage epithelial ovarian carcinoma had a better survival rate overall and survival free of progression along with adjuvant chemotherapy than those who did not receive it, according to four randomized control trials that examined chemotherapy which used platinum as its basis. The 2003 International Collaborative Ovarian Neoplasm Trial 1 (ICON1), however, found similar results in women with a greater risk of receiving adjuvant chemotherapy but not in the other studies. Given the high percentages of survival, surgical treatment alone is advised over adjuvant chemotherapy combined with vigilant monitoring in Stage 1A or Stage 1B epithelial ovarian cancer or Grade 1 endometrioid carcinomas [24]. Another prospective randomized Phase 3 trial was conducted. Endpoints were overall patient survival and survival of patients without recurrence. Patients were chosen on a random basis for either adjuvant chemotherapy using platinum as a basis or observation followed by surgery (recurrence-free survival). It demonstrated that chemotherapy increases overall survival along with no recurrence in people with residual disease who are not in an optimal staging. However, similar findings were not seen in patients who were in an optimal staging (people having some chances of residual disease). This shows that the micro-metastasis that is missed during surgical staging in early-stage ovarian cancer is impacted by adjuvant chemotherapy [25].

Platinum along with taxane are typically used in treating people suffering from ovarian carcinoma of advanced type. The best tumor debulking will determine if intravenous (IV) chemotherapy or intraperitoneal (IP) chemotherapy is an option. In contrast to the cohort receiving cisplatin and cyclophosphamide, diseased women receiving the combination of cisplatin and paclitaxel had increased overall survival, according to the Phase III trial of Gynecologic Oncology Group (Study 111) (GOG111). Platinum-based carboplatin or cisplatin and a member of the taxane family, such as docetaxel or paclitaxel, are the first-line chemotherapeutic agents for epithelial ovarian carcinoma. Numerous investigations have found that carboplatin equally shows its effect as cisplatin and also has a better tolerance. Additionally, compared to regular three-weekly chemotherapy, a third drug, or a lengthier treatment cycle, weekly dose-dense chemotherapy with a combination of carboplatin and paclitaxel has not shown any improved progression-free survival (PFS) [23]. Chemotherapeutic drugs are injected intravenously, intraperitoneally, or both. IP carboplatin treatment is safe for ovarian cancer patients who are older. Four seminal studies showed enhanced survival benefits of IP or IV chemotherapy, with good evidence to back the claim. Clinical application, however, has been patchy [26,27]. This is primarily because patients receiving IP chemotherapy experience more toxic side effects, particularly thrombocytopenia, neutropenia, neurotoxicity, and adverse gastrointestinal symptoms.

Differential diagnosis

The differential diagnosis for ovarian carcinoma include: 1) Carcinoma of the rectosigmoid colon and descending colon is the most common site of metastasis from ovarian carcinoma followed by ascending colon, 2) Adenocarcinoma of the gastric region, which happens because ovarian cancer mostly spreads along the peritoneum, 3) Cancer of the gastrointestinal portion, where the cancer spreads into the intestines and they adhere with each other and this cancer is metastatic, 4) Torsion of ovaries, where the tumor gets bigger in size with time and it starts to suppress the blood supply to the ovaries which causes their torsion, 5) Cysts in the peritoneum, where the peritoneum is affected in ovarian cancer as it tends to spread along the peritoneum, 6) Mass in the retro-peritoneal region, where tumor spreads into the retro peritoneal region and also starts to grow there, 7) Development of fibroids in the uterus, 8) Endometriosis, which can occur together with ovarian cancers, 9) Malignant tumors in the sweat glands, 10) Serous adenocarcinomas, 11) Adenocarcinomas, which are not differentiated, 12) Adenocarcinomas of small cells, and 13) Brenner tumors [28].

Prognosis

The stage of the disease when it was diagnosed has a direct effect on the prognosis of cancer of the ovary. Additionally, it has a strong correlation with baseline status of performance, FIGO stage, and the amount of illness that remains after cytoreductive surgery which must be primary [29]. Ovarian cancer patients had a median rate of survival of 40% to 50% at 10 years, with stage-related survival for Stage 1 patients ranging from 70% to 92% to Stage 4 patients at fewer than 6%. Five-year survival rates for women whose disease has expanded to nearby tissues fall to 80%, and to 25% for those whose disease has metastatic spread. Most patients with recurrent ovarian cancer experience a malignant intestinal obstruction in the advanced stages, which is very challenging to treat. The primary treatment for such patients is palliative symptom management. Debulking surgery is the best indicator of prognosis because overall survival and PFS are closely connected with the amount of post-operative illness that remains [30]. The prognosis of ovarian cancer is depicted in Table 1.

**Table 1:** Prognosis of ovarian cancer

	Invasive epithelial ovarian cancer	Ovarian stromal tumours	Ovarian germ cell tumours	Fallopian tube carcinoma
Stage 1	90%	95%	98%	87%
Stage 2	70%	78%	94%	86%
Stage 3	39%	65%	87%	52%
Stage 4	17%	35%	69%	40%

## Conclusions

Ovarian cancer is a leading cause of cancer incidence and deaths all over the globe in the female population. This review article describes various problems associated with ovarian carcinoma and summarizes epidemiological and etiological studies that have successfully identified the genetic, environmental, and lifestyle factors that can cause increment or decrease the risk of this lethally harmful disease. The genetic mutations in BRCA genes as well as increasing age are one of the most common risk factors of ovarian cancer. These factors have a great impact on the different patterns and trends related to ovarian cancer incidences and deaths seen all across the globe. The WHO classification of ovarian cancer has also been depicted in the article with brief histopathology from which we conclude that ovarian cancer is of various types. The most common type of ovarian cancer is the epithelial type and it can be found in about 90% of the affected people.This article has also focused on the different treatments that can be given to different patients at different stages of ovarian cancer. We conclude that initial treatments make use of cisplatin-based chemotherapy. The treatments are most effective if the cancer is detected in early stages. If the cancer is detected in later stages then the chemotherapy may not be that much effective and surgery is the only option. The article also shows comparison between the different treatment methods based on their effectiveness on different patients. Early warning signs to prevent the further advancement of this cancer in people have also been discussed. The most common sign is uterine bleeding even after menopause. We also conclude that chance of survival when ovarian cancer recurs is very less since treatment given does not have much needed effect on the patients health.
